# Channels of participation: Political participant types and personality

**DOI:** 10.1371/journal.pone.0240671

**Published:** 2020-10-29

**Authors:** David Johann, Markus Steinbrecher, Kathrin Thomas

**Affiliations:** 1 Library, Eidgenössische Technische Hochschule, Zürich, Switzerland; 2 Center for Military History and Social Sciences of the Bundeswehr, Potsdam, Germany; 3 Department of Politics and International Relations, University of Aberdeen, Aberdeen, United Kingdom; Aalborg University, DENMARK

## Abstract

This article employs a person-centred approach to test the relationship between personality traits and empirically defined political participant types. We argue that it is more appropriate to focus on types of participants to test the relationship between personality and political participation than on individual modes or latent dimensions of political participation. Our reasoning is that the person-centred approach allows us to learn more about how and why citizens combine different modes of participation from a tool kit of available political activities to achieve a goal as a function of their personality. We rely on data collected by the German Longitudinal Election Study 2017 (GLES, ZA6801). On the basis of a set of survey questions enquiring on political activities that people take part in, Latent Class Analysis allows us to identify three political participant types (inactives, voting specialists, and complete activists). The 10-item Big Five Inventory (BFI-10) measures respondents’ personality traits. Our findings suggest that conscientious people are more likely to affiliate with the voting specialists and extroverts with the more active participant types in Germany.

## 1 Introduction

An extensive and increasing body of literature studies the way in which citizens engage in politics and what might explain differences in their participatory patterns [1–10]. While a majority of these studies focus on individual modes of participation, such as voting, attending demonstrations, or signing petitions, more recent research has emphasised the need to re-consider the ways in which we think about political engagement suggesting to empirically identify either activity- or person-centred classifications: The activity-centred perspective views participation in different dimensions of activity types. For example, studies distinguish formal from informal, online from offline, legal from illegal forms of engagement using factor analysis and related methods [11–15]. The person-centred perspective focuses on citizens combining a variety of individual channels of participation from a virtual toolbox, arguing that citizens assess the best available participatory options and select those tools that they deem to be most effective to achieve their goals [3, 8, 13, 16–25].

For emphasis, to shed light on how the person-centred perspective differs from investigating individual modes of participation or employing an activity-centred approach and why it is important to study participation in this way, we borrow from the environmental awareness literature: At the individual level, environmental awareness can be measured with numerous indicators, such as usage of public transportation. It is possible to investigate which individual variables (e.g., gender, age, personality traits) drive the use of public transport exploring their effect on this single item. The researcher’s research interest in this example is to explain why citizen chose public transportation. However, environmental awareness might also include items like car usage, cycling, meat consumption and recycling, and some of these indicators may describe the same latent dimension of environmental awareness [26]. The frequency of using public transport, a car or a bike may represent the same latent construct “choice of means of transportation for environmental reasons”. Indeed, it might be desirable to investigate a broader question focusing on the choice of means of transportation for environmental reasons and how gender, age, and personality traits are related to this latent dimensions. This activity-centred perspective allows answering whether and how different latent dimensions of activities are used. However, neither approach reveals adequately why citizens opt different activities simultaneously, i.e., choose particular means of transportation, but also abstain from eating meat to protect environment, and what might explain this. The person-centred approach achieves this by using cluster-analytical methods, such as Latent Class Analysis (LCA), which allows identifying relatively homogeneous groups of citizens who represent certain types of environmentally aware people: For example, the completely unaware, somewhat environmentally conscious people who may select certain green means of transportation, and very environmentally aware citizens, who use green means of transport but might also stay away from eating meat, recycle frequently etc.

All approaches should be seen as complementary rather than competing, as they offer different perspectives on participation [8, 13, 20]. Depending on the research question and focus, one or the other way of viewing participation may be more appropriate. As this article is interested in uncovering how personality is related to combining several different modes of participation to achieve a goal, we employ a person-centred approach. This is appropriate, because we are interested in individuals’ traits and behaviours and assume that personality is reflected in their participatory behaviour.

A large number of studies has emphasized the role of personality traits in explaining why people employ different, individual modes of engagement [27–39]. For example, these studies show that open, conscientious, and extroverted people are frequently more vocal about politics and more likely to take part in political activities, while other traits have produced inconsistent results. However, the link between personality traits and citizens combining different individual modes of political participation from their available toolbox has not been studied yet.

Thus, this article contributes to the broader literature by asking how citizens combine different modes of political participation as a function of their personality. We focus on the German case, as the country is traditionally characterised by high levels of political participation in elections, petitions, demonstration etc. [13, 40] In addition, the German Longitudinal Election Study collected high quality data suitable to conduct LCA. As such, the article moves beyond thinking about participation in simple ways and investigates the more complex, underlying patterns of political participation among various groups of citizens in Germany.

We begin by discussing the previous literature on political participant types and personality. Next, we present our data and methods. Our results section provides insights to the empirically defined political participant types, then moves on to the effects of personality on the affiliation with these types of participants. We close with a discussion of our results and conclusion outlining the implications for future research.

## 2 Political participant types and personality

Reviving the person-centred idea of political participant types [3, 8], scholars have started employing empirical solutions to structure how citizens combine their available channels of participation. This approach is arguably useful to study the facets of political activism, especially given that an increasing number of individual modes is available in citizens’ tool kits [8, 19–24]. Combining different modes from a tool kit of political activities might be more promising for citizens to successfully influence politics than to be politically active in only one way [18–22].

The number of empirically established political participants types may depend on various factors including differences in question wording and focus, methodology, but also the political and cultural context. Yet, most typologies find at least three types of participants: inactives, voting specialists, and complete activists [8, 13, 19, 22]. We refer to the language used by [8], but acknowledge that some of these types have been referred to using different terms. Any additional types of political participants mentioned in previous literature typically cluster around a level of specialisation [13, 17, 20, 21, 23, 24]. Some scholars only report engaged and disengaged participators [23, 24], others all-round activists, high-voting engaged, mainstream and disengaged participants [22, 41], or agitators, outsiders, activists, and conventionals [16]. A study on youth online participation identifies engagers and non-engagers [42].

While the literature on individual modes of participation has identified a variety of reasons why people are politically active [1–10, 13, 43, 44], little research investigated why citizens combine activities resulting in participant typologies [16, 19–25]. Links have been established between age, socio-economic status, education, and political knowledge [16, 19, 23, 41]. Furthermore, recent research suggests that political interest appears to be the principal driver for youth engagement [42]. Factors beyond the traditional socio-economic model plus political involvement have not been studied in relation to participant typologies yet.

Social psychology suggests that personality traits have the potential to influence citizens’ social and political behaviour, as the different traits are activated and stimulated in interaction with the social environment [45]. It is believed to shape citizens’ cognitive, emotional, and behavioural responses, which, in turn, might influence whether people adopt politically relevant attitudes or behaviour [29]. Personality is often measured in five dimensions: Openness to experience, Conscientiousness, Extraversion, Agreeableness and Neuroticism, also known as the the Big Five or OCEAN model of personality. We outline which attributes are typically associated with these dimension in Table 1 [30, 31, 33, 46], acknowledging that different empirical measures may only capture some of these facets (e.g., the BFI-10 measure discussed in the Data and Methods Section).

**Table 1:** Personality traits.

Personality trait	Characteristics
**Openness (O)**	Tolerance, creativity, interest, originality, and curiosity
**Conscientiousness (C)**	Ambition, hard-work, thorough-ness, planning, and goal-orientation
**Extraversion (E)**	Open-mindedness, activity, energy, friendliness, assertive-ness, and talkativeness
**Agreeableness (A)**	Generosity, empathy, communal orientation, and altruism
**Neuroticism (N)**	Uneven-temperateness, restlessness, and irrationality

Prior research provided evidence that personality traits affect individual modes of participation [32]. Especially open, conscientious, and extroverted people were found to be more actively involved in various kinds of political activities [27, 30, 31, 33–35, 38, 47, 48]. Findings for agreeableness and neuroticism are inconsistent, however [27–31, 34, 35, 38, 47–50]. While personality has strong predictive power when individual participation items are concerned, an account of how it affects participant typologies is still outstanding. We would expect that certain personalities frequently embrace the same channels of participation, as combined in the political participant types. Following previous research on personality and individual modes [35, 38], we posit that openness to experience and extraversion should be related to the affiliation with the more active political participant types (i.e., the Complete Activists or possible other activists that frequently embrace modes beyond voting). In turn, especially introversion and a lack of openness may explain why citizens do not affiliate with participant types that participate politically beyond voting [7, 13]. Moreover, thinking about agreeableness, we would expect that individuals scoring high on this trait may be less likely to belong to the more active participant types: collective action, which is inevitably included in all kinds of political activity beyond voting, comprises an increased conflict potential that agreeable persons are eager to avoid [4, 35, 51]. With regard to conscientiousness, we assume that conscientious citizens do what is expected of them, but do not necessarily engage more than that. For instance, citizens are expected to make use of their right to participate in elections [13, 52], but there is no expectation to be involved in other modes of political participation to the same extent, even though this might be desirable from a normative point of view [43]. Thus, conscientious citizens may affiliate with the voting specialists—at least as long as the costs of participation remain relatively low [5, 6]—but may not embrace other ways to participate beyond voting. Finally, we can only assume that emotionally unstable people are, in general, politically inactive, as they are unlikely to thrive in any political task.

H1: Open individuals are more likely to affiliate with the complete activists in comparison with the other participant types.
H2: Extroverts are more likely to affiliate with the complete activists in comparison with the other participant types.
H3: Agreeable persons are more likely to affiliate with the voting specialists in comparison with the other participant types.
H4: Conscientious individuals are more likely to affiliate with the voting specialists in comparison with the inactives.
H5: Emotionally unstable individuals are more likely to affiliate with the inactives in comparison with the other participant types.

## 3 Data and methods

We analyse cross-sectional, post-election data collected by the German Longitudinal Election Study [53]. The sample is representative of Germany’s population aged 16 and above. For analysis, we restrict the sample to the population eligible to vote, i.e., citizens aged 18 and above. The reason for this is that voting is one core political activity included in our typology of political participants. Fieldwork took place between 26 September and 30 November 2017 and was completed by Kantar, Germany, and infratest dimap using Computer Assisted Personal Interviewing. Cash incentives were provided conditional on the completion of the interview. The survey achieved a response rate of 29.4 percent.

Among other things, the GLES survey included questions on political participation and personality. Respondents were asked to indicate whether they (1) participated in a demonstration, (2) actively took part in public discussions, (3) donated to a political party, (4) signed a petition (conventional petition or e-petition), and (5) attended an election campaign in the past 12 months. In addition, the survey enquired whether or not respondents voted in the 2017 national election. All participation items were captured by a dichotomous no/yes-response code.

Personality was measured using the BFI-10 [54–56]. The Big Five framework of personality is arguably the most adequate instrument to measure personality across cultures and countries [46, 57, 58], but other proposals to measure personality exist [59–62]. Recent studies have suggested that the Big Five come with a few problems, such as masking effects of individual traits or context sensitivity [63, 64]. As the BFI-10 have repeatedly been validated and verified in the German context [54–56], we are confident that we are measuring a valid concept.

The Big Five variables were coded in a way that higher values indicate extroverted, open, agreeable, conscientious, and emotionally unstable individuals. A Principal Component Factor Analysis revealed that the 2x5 indicators can be assigned to the five personality dimensions as expected (see S1 Table). For each dimension we thus calculated a sum index of the two complementary items for each of the Big Five dimensions ranging from 0 to 8. Correlations of the five dimensions are presented in S2 Table.

The participant types were constructed employing LCA [20–24] using the poLCA package in R [65]. We estimated a latent class regression model, which simultaneously permitted us to include independent variables into the model, predicting the probability of affiliating with a specific type of participant [65]. As control variables, we included civic attitudes (civic duty, interest in politics, political knowledge, internal and external efficacy) as well as some socio-demographic characteristics (education, age, gender). Higher values on the civic attitude indicators correspond with a high level of civic duty, political interest and knowledge, internal and external political efficacy (see S3 Table).

Although income has sometimes been identified as a driver of participation, we do not include this variable in our empirical model, because the indicator suffers from high item non-response. This is not a surprise, as obtaining accurate reports of income in Germany is traditionally somewhat difficult and prone to error depending on the sample design, the representation of particular groups, income measurement and social desirability concerns [66, 67].

By including all other control variables mentioned above, our estimated effects of the Big Five on the affiliation with different types of participants are fairly conservative, because the effects of personality on political participation are often indirect in nature, potentially mediated by some of our control variables [32, 38]. For reasons relating to clarity and ease of interpretation, we present a relatively parsimonious model. However, we have calculated further model specifications with additional control variables, such as democracy satisfaction and religiosity to validate our results. When we include these controls, we find that neither of the variable has a significant effect and the Big Five reveal similar effects on the affiliation with our political participant types. These additional results support our findings presented below.

## 4 Political participant types in Germany

We present the results of the LCA in Table 2 (for a detailed report on class probabilities, see S4 Table). We identify inactives (10.7 percent), voting specialists (64.7 percent), and complete activists (24.6 percent). This corresponds with the previous literature on participant typologies [8, 13]. The Akaike and Bayesian Information Criteria confirm that this three-class solution is the best fit for the data (see S5 Table). While the inactives tend to refrain from all modes of participation, the voting specialists are likely to take part in elections but display small levels of activism elsewhere. The complete activists are likely to employ most channels of participation.

**Table 2:** Probability to participate by participant type.

	Inactives	Voting Specialists	Complete Activists
**Turnout**	17.66	99.14	98.68
**Demonstration**	1.23	0.44	31.07
**Petition**	8.98	12.26	62.41
**Donation**	0.00	1.34	19.33
**Discussion**	1.48	0.74	24.37
**Campaign**	0.00	0.39	13.20

## 5 Political participant types and personality in Germany

The results from the latent class regression model are presented in Table 3, which displays the impact of the independent variables on the probability of affiliating with a specific participant type. The inactives serve as the reference category in the first and second column of Table 3; the voting specialists serve as the reference category in the third column of Table 3.

**Table 3:** Latent class regression model pesults.

	Inactives vs. Voting Specialists	Inactives vs. Complete Activists	Voting Specialists vs. Complete Activists
**Openness**	-0.045	0.033	0.078
	(0.074)	(0.089)	(0.058)
**Consciousness**	0.077	-0.280*	-0.357**
	(0.084)	(0.101)	(0.709)
**Extraversion**	-0.013	0.259**	0.272**
	(0.065)	(0.085)	(0.060)
**Agreeableness**	-0.024	-0.046	-0.021
	(0.079)	(0.098)	(0.070)
**Neuroticism**	0.128	0.188	0.059
	(0.076)	(0.094)	(0.063)
**Civic Duty**	0.811**	0.809**	-0.002
	(0.156)	(0.161)	(0.090)
**Political Interest**	0.293	1.160**	0.867**
	(0.154)	(0.200)	(0.140)
**Knowledge Actors**	0.635**	1.041**	0.406*
	(0.165)	(0.225)	(0.168)
**Knowledge System**	0.507*	0.580*	0.073
	(0.180)	(0.213)	(0.140)
**Internal Efficacy**	0.099	0.187	0.088
	(0.130)	(0.160)	(0.106)
**External Efficacy**	0.017	0.079	0.062
	(0.118)	(0.157)	(0.116)
**Education**	0.623	1.898**	1.275**
	(0.333)	(0.379)	(0.214)
**Age**	0.012	0.002	-0.010
	(0.008)	(0.009)	(0.006)
**Women**	0.375	0.395	0.020
	(0.276)	(0.332)	(0.213)
**Constant**	5.422**	-11.037**	-5.615**
	(1.557)	(1.783)	(1.120)
***n***	1,884	1,884	1,884

The dependent variable is the affiliation with a specific type of political participant. Standard error in parentheses.

** p-value < 0.01,

* p-value < 0.05.

The results suggest that personality has no significant effect on the affiliation with the voting specialists in comparison with the inactives. Only a higher sense of civic duty (Coef. = 0.811, p-value < 0.01) as well as knowledge about political actors (Coef. = 0.635, p-value < 0.05) and the system (Coef. = 0.507, p-value < 0.1) reveal statistically significant positive effects.

The results in the second column of Table 3 indicate that extroverts (coef. = 0.259, p-value < 0.01) are more likely and conscientious people (coef. = -0.280, p-value < 0.1) are less likely to affiliate with the complete activists in comparison with the inactives. In addition, the results reveal that a high sense of civic duty (coef. = 0.809, p-value < 0.01), political interest (coef. = 1.160, p-value < 0.01) as well as knowledge of political actors (coef. = 1.041, p-value < 0.01) and the system (coef. = 0.580, p-value < 0.1) matter. Unsurprisingly, well educated people are also more likely to affiliate with the complete activists in comparison with the inactives (coef. = 1.898, p-value < 0.01).

Similar results are revealed when looking at the effects on the affiliation with the complete activists in comparison with the voting specialist: Extroverts (coef. = 0.272, p-value < 0.01) are more likely and conscientious people (coef. = -0.357, p-value < 0.01) are less likely to affiliate with the complete activists. In addition, political interest (coef. = 0.867, p-value < 0.01), knowledge about political actors (coef. = 0.406, p-value < 0.1) and education (coef. = 1.275, p-value < 0.01) reveal statistically significant positive effects.

**Fig 1:**
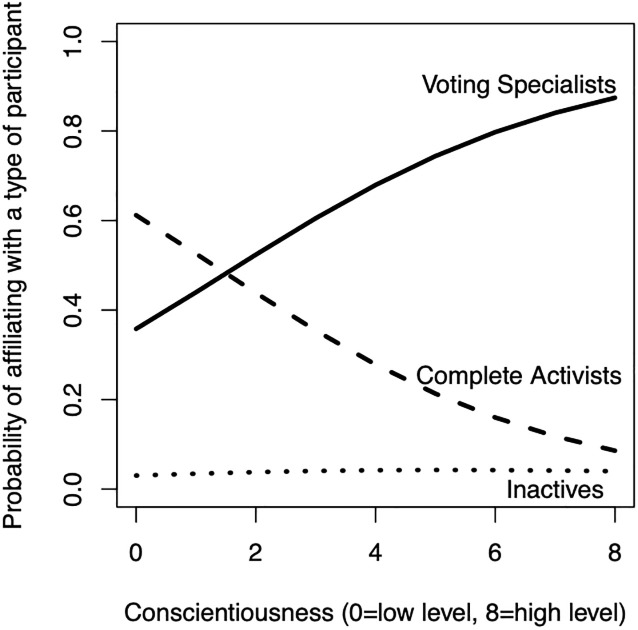


Fig 1 presents the predicted probabilities to affiliate with a specific participant type by level of conscientiousness. The plot shows that the higher people score on the conscientious measure, the more likely they are to affiliate with the voting specialist (solid line). The likelihood of affiliating with the complete activists appears to decrease with higher levels of conscientiousness (dashed line).

**Fig 2:**
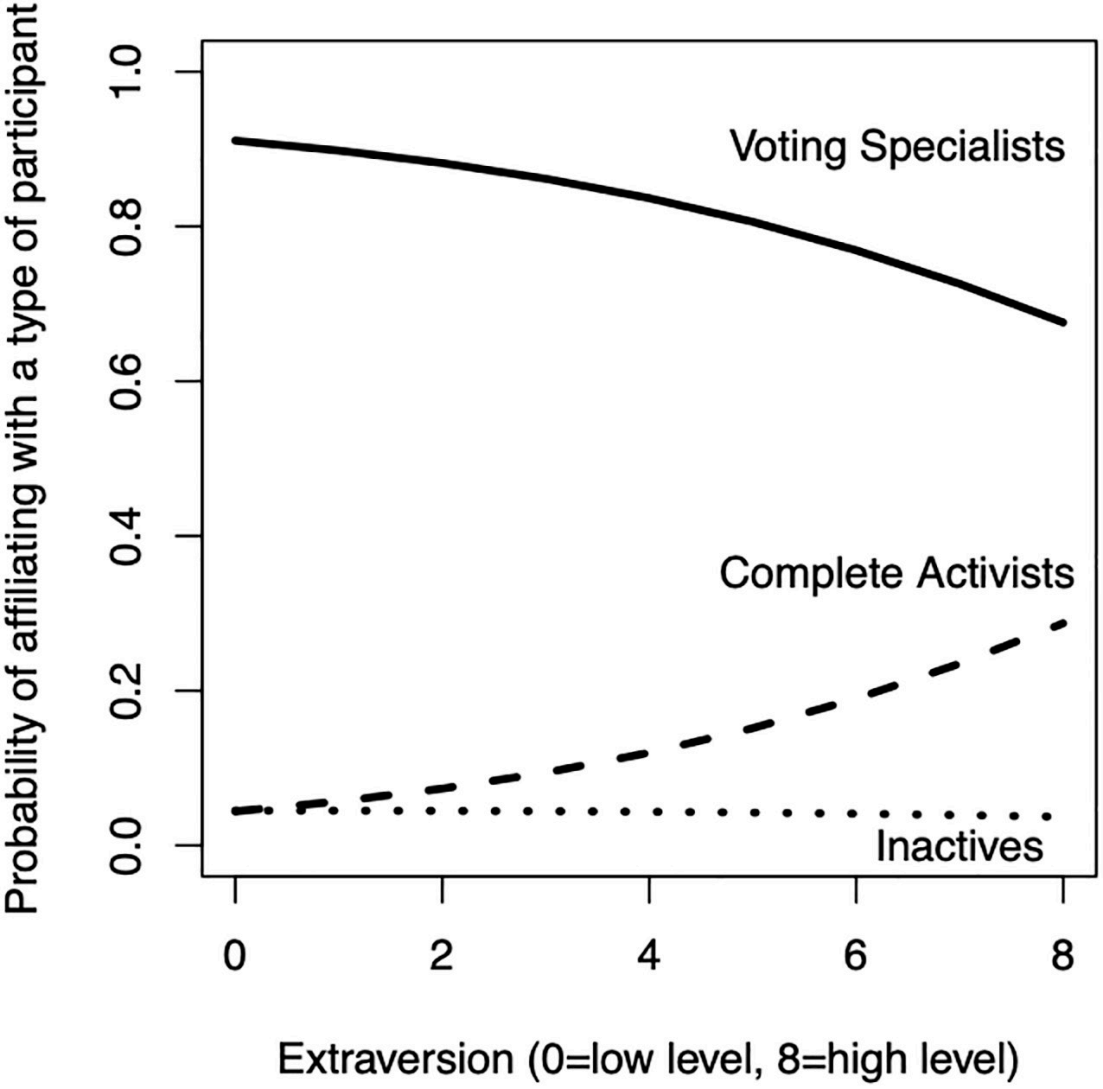


Fig 2 presents the predicted probabilities to affiliate with a specific participant type by level of extraversion. The likelihood of affiliating with the complete activists increases with a higher score on extraversion (dashed line), while the likelihood of belonging to the voting specialist decreases (solid line).

## 6 Discussion and conclusion

This article empirically estimated a typology of political participant types in Germany following examples from the previous literature [8, 13, 20–23]. In line with prior findings [8, 13], our results suggest a three-class solution of inactives, voting specialist, and complete activists. Looking at political participation through the lenses of political participant types is beneficial assuming that citizens select and combine various channels of participation from their available tool kit to achieve a particular goal [18, 20–22].

We also investigated whether the affiliation with the different participant types is a function of citizens’ personality. The results suggest that personality indeed predicts the likelihood of affiliating with a particular type of participant. For instance, the more extroverted people are, the higher is the likelihood of affiliating with the most active type. Moreover, the more conscientious people are, the higher is the likelihood that they affiliate with the the voting specialists. This supports our hypothesis with regard to these traits. However, we do not find supporting evidence for the remaining personality traits of the Big Five inventory, i.e., openness, agreeableness and neuroticism. While we are surprised about the lack of evidence for openness, which reliably predicts why people employ individual modes of participation, the null results for agreeableness and neuroticism may be less surprising, given that prior research on individual modes of political participation found conflicting results.

Of course, our analyses could not include all possible factors that may explain the affiliation with these political participant types. It is possible that additional factors, such as citizens’ personal political communication about politics, their social capital, value and ideological orientations, play a crucial role. For example, these aspects may speak to the level of altruism of political engagement, which could trigger the trait of agreeableness. Future research should consider these factors when studying political participant types and personality.

While this article focused on the direct impact of personality on the affiliation with political participant types, prior research has also suggested that personality traits may lead to the acquisition of other politically relevant attitudes or behaviour, i.e., other attitudes may mediate the effect of personality traits [29, 32–38]. As this study provides an initial account of the relationship between personality and participant typologies and test whether people with certain personalities frequently combine the same activities to achieve their goal at all, we do not conduct mediation analysis. By all means, we would recommend that future work considers potential indirect influences of personality on the affiliation with different types of participants.

It is also noteworthy that this study looked at one case at a specific point in time: Germany in 2017. The country is characterised by high turnout rates, an increasing number of citizens engagement in petitions and e-petitions as well as topical demonstrations for different causes. Comparative research might be desirable to confirm our results for other democracies and should be able to evaluate whether our findings are typical for (Western) democracies in general.

In conclusion, learning more about how people combine political activities to achieve a goal and also what kinds of personalities affiliate with different participants types is important for the scholarly literature and beyond. The academic field will be interested in the patterns of representation and more generally who and how different types take part in politics. However, one practical implication could be that campaigners might employ this information to design campaigns that are more inclusive and aim to activate and motivate those who might be less likely to actively engage. Of course, this requires access to relevant data on personality traits. While these could be difficult to obtain, projects, such as the BBC and University of Cambridge Personality Project [68], have made an attempt to collect such data on a larger scale.

## Supporting information

S1 Table Rotated factor loadings of the Big Five items (OCEAN) using a principal-component factor analysis (Varimax). (PDF)

S2 Table Correlations Big Five dimensions (OCEAN). (PDF)

S3 Table Summary statistics. (PDF)

S4 Table Average latent class probabilities. (PDF)

S5 Table Model fit statistics. (PDF)
