# Supplementary material for: Channels of participation: Political participant types and personality
Source: PLoS One. 2020 Oct 29;15(10):e0240671. doi: 10.1371/journal.pone.0240671 (PMC7595324; doi:10.1371/journal.pone.0240671)
Supplement: S1 Table — (PDF) [file pone.0240671.s001.pdf]

Table S1. Rotated Factor Loadings of the Big Five Items (OCEAN) using a Principal-Component Factor Analysis (Varimax)

	**O**	**C**	**E**	**A**	**N**
**O1**	0.8374	-0.0152	0.0397	0.0012	-0.0889
**O2**	0.7568	0.2080	0.0928	0.1586	-0.0452
**C1**	0.0478	0.7819	0.0730	0.0041	-0.0849
**C2**	0.0853	0.8164	-0.0344	-0.0080	-0.0381
**E1**	0.2821	0.1095	0.7351	0.0237	-0.0610
**E2**	-0.0601	-0.0456	0.8520	0.0332	-0.0113
**A1**	0.1528	-0.0568	0.0718	0.8608	0.0713
**A2**	-0.3665	0.2561	-0.1141	0.5391	-0.2146
**N1**	0.0668	0.0064	-0.0407	-0.0717	0.8641
**N2**	-0.3009	-0.1665	-0.0152	0.1602	0.7425

Note: Eigenvalues of the factors between 1.002 and 2.085. The five factors explain 68,15 percent of the total variance observed.

Question wording: Please tell me for each of the following statements, using this scale ([1] Does not apply at all, [2] Rather does not apply, [3] Neither applies nor does not apply, [4] Rather applies, [5] Applies completely), to what extent they apply to you: I tend to be somewhat shy and reserved (O1); I easily trust other people and see the good in others (A1); I perform tasks very thoroughly (C1); I am relaxed and can handle stress well (N1); I have an active imagination and am inventive (E1); I am outgoing and sociable (O2); I tend to find fault with others (A2); I tend to be lazy (C2); I easily get nervous and uneasy (N2); I have few artistic interests (E2).
