# Supplementary material for: Channels of participation: Political participant types and personality
Source: PLoS One. 2020 Oct 29;15(10):e0240671. doi: 10.1371/journal.pone.0240671 (PMC7595324; doi:10.1371/journal.pone.0240671)
Supplement: S2 Table — (PDF) [file pone.0240671.s002.pdf]

Table S2. Correlations Big Five Dimensions (OCEAN)

	**C**	**E**	**A**	**N**
**O**	0.160**	0.201**	-0.013	-0.176**
**C**		0.042	0.097**	-0.191**
**E**			0.017	-0.078**
**A**				-0.022

Note: ** p-value $<$ 0.01, * p-value $<$ 0.05.
