# Supplementary material for: Channels of participation: Political participant types and personality
Source: PLoS One. 2020 Oct 29;15(10):e0240671. doi: 10.1371/journal.pone.0240671 (PMC7595324; doi:10.1371/journal.pone.0240671)
Supplement: S3 Table — (PDF) [file pone.0240671.s003.pdf]

Table S3. Summary Statistics

Statistic	N	Mean	St. Dev.	Min	Max
**Turnout**	1884	1.90	0.30	1	2
**Demonstration**	1884	1.08	0.27	1	2
**Donation**	1884	1.06	0.23	1	2
**Discussion**	1884	1.07	0.25	1	2
**Campaign**	1884	1.04	0.18	1	2
**Petition**	1884	1.24	0.43	1	2
**Openness**	1884	4.87	1.76	0	8
**Consciousness**	1884	6.13	1.52	0	8
**Extraversion**	1884	5.07	1.79	0	8
**Agreeableness**	1884	4.77	1.48	0	8
**Neuroticism**	1884	3.07	1.71	0	8
**Civic Duty**	1884	4.15	1.21	1	5
**Political Interest**	1884	3.26	0.98	1	5
**Knowl. Actors**	1884	2.33	0.88	0	3
**Knowl. System**	1884	1.31	0.77	0	2
**Internal Efficacy**	1884	3.06	1.14	1	5
**External Efficacy**	1884	2.48	0.91	1	5
**Education**	1884	0.44	0.50	0	1
**Age**	1884	51.27	18.20	18	94
**Women**	1884	0.47	0.50	0	1
