# Supplementary material for: Channels of participation: Political participant types and personality
Source: PLoS One. 2020 Oct 29;15(10):e0240671. doi: 10.1371/journal.pone.0240671 (PMC7595324; doi:10.1371/journal.pone.0240671)
Supplement: S4 Table — (PDF) [file pone.0240671.s004.pdf]

Table S4. Average Latent Class Probabilities

	**Inactives**	**Voting Specialists**	**Complete Activists**
**Inactives**	0.9256	0.0594	0.0149
**Voting Specialists**	0.0252	0.8982	0.0766
**Complete Activists**	0.0033	0.1326	0.8641
