# Supplementary material for: Channels of participation: Political participant types and personality
Source: PLoS One. 2020 Oct 29;15(10):e0240671. doi: 10.1371/journal.pone.0240671 (PMC7595324; doi:10.1371/journal.pone.0240671)
Supplement: S5 Table — (PDF) [file pone.0240671.s005.pdf]

Table S5. Model Fit Statistics

	AIC	BIC
**2 Classes**	5893.048	6042.659
**3 Classes**	5647.258	5913.233
**4 Classes**	5689.258	6071.597
